# 活化部分凝血活酶时间和D-二聚体在慢性淋巴细胞白血病中的预后价值

**DOI:** 10.3760/cma.j.issn.0253-2727.2021.05.013

**Published:** 2021-05

**Authors:** 进 毛, 必慧 潘, 华 尹, 佳竹 吴, 奕 夏, 莉 王, 建勇 李, 卫 徐

**Affiliations:** 南京医科大学第一附属医院（江苏省人民医院）血液科 210029 Department of Hematology, the First Affiliated Hospital of Nanjing Medical University, Nanjing 210029, China

慢性淋巴细胞白血病（CLL）国际预后指数（CLL-IPI）是免疫化疗时期预测CLL预后的标准模型[1]。此外，许多基因突变如NOTCH1、SF3B1和BIRC3也被证实与不良预后相关[2]–[3]。但由于经济和技术上的限制，许多分子和基因的检测在常规临床实践中无法正常开展。肿瘤患者的高凝状态已引起广泛关注，恶性肿瘤患者静脉血栓发生的风险大约是普通人群的7倍，而血液系统肿瘤患者发生静脉血栓的风险更高[4]。因此，及时发现癌症患者的高凝状态，尽早预防或治疗极其重要。目前国内尚缺乏异常血浆凝血参数与CLL患者预后相关性的研究。本研究回顾性分析了本中心329例初诊CLL患者凝血相关参数与预后的相关性。

## 病例与方法

1. 病例：回顾性分析2009年1月至2018年6月南京医科大学第一附属医院（江苏省人民医院）血液科329例初诊CLL患者临床资料。CLL的诊断参考2008年修订的国际CLL工作组（iwCLL）-国家癌症研究所（NCI）的标准[5]。排除标准：①确诊前曾患凝血系统相关疾病，如静脉或动脉血栓栓塞、肺栓塞或弥散性血管内凝血（DIC）；②1个月内接受过促凝或抗凝治疗；③合并其他类型的恶性肿瘤；④基线数据不完整。

2. 治疗方案：在符合条件的329例患者中，有199例（60.5％）接受了治疗，其中117例（58.8％）患者接受了FCR方案（氟达拉滨+环磷酰胺+利妥昔单抗）或FC方案（氟达拉滨+环磷酰胺）；17例（8.6％）患者接受苯达莫司汀治疗；55例（27.6％）患者接受苯丁酸氮芥联合利妥昔单抗或苯丁酸氮芥单药治疗；10例（5.0％）患者接受其他治疗方案，如伊布替尼、大剂量甲泼尼龙±利妥昔单抗。

3. 临床资料收集：收集患者的基线临床资料及相关实验室数据。此外，还收集了包括TP53状态、免疫球蛋白重链可变区（IGHV）基因突变状态、del（11q）和CD38等预后参数。TP53异常指TP53缺失和（或）突变，IGHV是否突变的临界值为98％（与胚系基因序列的一致性≥98％定义为IGHV无突变）。CD38则通过流式细胞术检测，阳性的临界值为30％。凝血相关指标包括活化部分凝血活酶时间（APTT）、D-二聚体（DD）、纤维蛋白原（FIB）、凝血酶原时间（PT）、PT国际标准化比值（PT-INR）和凝血酶时间（TT）。检测方法为收集外周血放入柠檬酸钠试管中，然后以3000 r/min的速度离心10 min分离血浆，用Sysmex CA-7000自动凝血分析仪（日本Sysmex公司产品）检测凝血参数。

4. 随访：随访截止时间为2020年1月，中位随访时间为63（5～140）个月。至首次治疗时间（TTFT）指自确诊之日至一线治疗开始的时间。总生存（OS）时间指自确诊之日至死亡或随访截止的时间。

5. 统计学处理：使用X-tile软件判断6个凝血相关指标的最佳截断值。通过IBM SPSS 21.0和GraphPad Prism 6.0软件分析数据。采用Kaplan-Meier曲线进行预后生存分析，Cox回归模型进一步分析各因素的独立预后价值，应用受试者工作特征（ROC）曲线和曲线下面积（AUC）比较不同预后模型的预后价值。*P*<0.05为差异有统计学意义。

## 结果

1. 临床特征：329例CLL患者男女比例2∶1，中位年龄59岁。85例（25.8％）有B症状，24.3％有贫血，31.0％有血小板减少，18.2％有白蛋白减少，21.6％有LDH增高，45.0％有β_2_-微球蛋白（β_2_-MG）水平增高。69.0％为Binet B或C期，23.6％有TP53异常，38.3％ IGHV无突变，14.0％存在del（11q），24.0％为CD38表达阳性。

利用X-tile软件我们计算出PT、PT-INR、APTT、FIB、TT和DD的最佳截断值分别为12.6 s、1.1、31.3 s、4.0 g/L、19.8 s和1.2 mg/L，根据上述临界值，高水平PT、PT-INR、APTT、FIB、TT和DD患者分别有70例（21.3％）、66例（20.1％）、44例（13.4％）、33例（10.0％）、41例（12.5％）和46例（14.0％）。

2. 生存预后分析：截至2020年1月，中位随访63（5～140）个月。Kaplan-Meier生存分析显示，高、低水平APTT组的中位TTFT分别为0个月和13个月（*P*＝0.047），中位OS时间分别为42个月与未达到（*P*<0.001）。高、低APTT组的5年TTFT率分别为（12.7±5.2）％和（33.2±2.9）％。高、低APTT组的5年OS率分别为（60.9±7.4）％和（79.5±2.5）％。高、低DD组的中位TTFT分别为2个月与12个月（*P*＝0.008），中位OS时间分别为71个月与未达到（*P*＝0.001）。高、低DD组的5年TTFT率分别为（14.3±5.8）％和（33.0±2.9）％，5年OS率分别为（56.3±8.0）％和（76.4±2.7）％。因此，APTT和DD水平较高与TTFT、OS更差相关。单因素分析结果示，APTT>31.3 s与TTFT［*HR*＝1.857，95％ *CI*（1.306～2.640），*P*＝0.001］和OS［*HR*＝3.882，95％ *CI*（2.476～6.086），*P*<0.001］较差相关，DD>1.2 mg/L也与TTFT［*HR*＝1.507，95％*CI*（1.071～2.121），*P*＝0.024］和OS［*HR*＝2.265，95％*CI*（1.407～3.648），*P*＝0.002］较差相关。

3. 亚组分析：为进一步探讨高水平APTT、DD与CLL预后的相关性，我们根据CLL-IPI评分中的危险因素（年龄、Binet分期、β_2_-MG水平、TP53状态和IGHV突变状态）进行了亚组分析。按年龄（≤65岁，>65岁）、Binet分期（A期，B、C期）、β_2_-MG水平（≤3.5 mg/L，>3.5 mg/L）和IGHV突变状态（突变，未突变）分组，高水平APTT患者的OS时间均较低水平APTT患者缩短（*P*值均<0.001）。但在TP53异常组，APTT水平对OS时间无影响（*P*＝0.804）。在年龄≤65岁（*P*＝0.005），Binet B、C期（*P*＝0.001），β_2_-MG≤3.5 mg/L（*P*＝0.004），TP53正常（*P*＝0.007）和IGHV未突变（*P*＝0.034）亚组，高水平DD与较差的OS相关。

4. 凝血系统指数（CSI）：我们将单因素分析中*P*<0.05且相互独立的凝血相关指标纳入Cox回归模型的多因素分析，结果显示，APTT和DD均为影响CLL患者TTFT和OS的独立因素（TTFT：*P*值分别为0.001、0.021；OS：*P*<0.001，*P*＝0.009）。根据APTT和DD对OS的危险比和回归系数（*β*），高APTT［*HR*＝3.785，95％*CI*（2.407～5.952），*β*＝1.331］和高DD［*HR*＝2.157，95％*CI*（1.335～3.484），*β*＝0.769］的权重分别为2和1。根据CSI将患者分为四组：CSI＝0（248例），CSI＝1（37例），CSI＝2（35例），CSI＝3（9例）。Kaplan-Meier曲线分析显示，CSI得分越高，患者的OS时间越短（*P*<0.05），且可显著区分任何两个相邻的风险群体（图1A）。而CSI＝1组和CSI＝2组间TTFT的差异无统计学意义（*P*＝0.330），且CSI＝3亚组病例数较少，于是在TTFT分析中，我们将患者分为两个危险组（CSI＝0、CSI＝1～3），结果显示，两组间未开始首次治疗患者比例的差异无统计学意义（*P*＝0.003）（图1B）。将CSI和CLL-IPI中的预后因素纳入Cox回归模型的多因素分析中，结果提示，CSI>1为TTFT（*P*＝0.021）和OS（*P*<0.001）的独立危险因素（表1）。

**表1:** 影响慢性淋巴细胞白血病患者至首次治疗时间（TTFT）和总生存（OS）的多因素分析

因素	TTFT		OS	
	*HR*（95％ *CI*）	*P*值	*HR*（95％ *CI*）	*P*值
年龄>65岁	0.705（0.512～0.970）	0.032	1.198（0.725～1.979）	0.481
BinetB或C	2.285（1.570～3.326）	<0.001	2.156（1.044～4.455）	0.038
β_2_-MG>3.5mg/L	1.767（1.261～2.328）	<0.001	1.739（1.017～2.972）	0.043
TP53异常	1.713（1.261～2.328）	0.001	1.925（1.198～3.095）	0.007
IGHV未突变	1.450（1.070～1.964）	0.016	2.389（1.404～4.064）	0.001
CSI>1	1.643（1.079～2.501）	0.021	3.848（2.254～6.572）	<0.001

注：β_2_-MG：β_2_-微球蛋白；IGHV：免疫球蛋白重链可变区；CSI：凝血系统指数

**图1:**
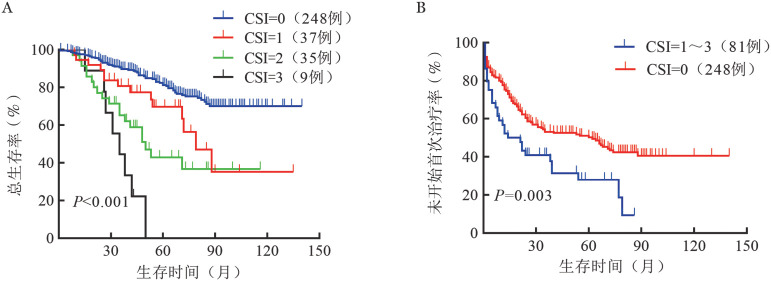
不同凝血系统指数（CSI）慢性淋巴细胞白血病患者的总生存（A）和未开始首次治疗（B）曲线

5. CLL-IPI与CSI组合构建新型预后评分体系：我们将CLL-IPI与CSI组合构建新的预后评分模型并通过ROC曲线和AUC评估预测精度。结果显示，对于OS，CSI联合CLL-IPI较单纯CLL-IPI的AUC值更高且差异具有统计学意义（0.783对0.761，*P*＝0.002），而对于TTFT的预测，差异则无统计学意义（0.773对0.767，*P*＝0.800）。

## 讨论

CLL患者的临床过程呈高度异质性，新药或新的治疗策略已经可以克服或部分克服一些既往不良预后因素，但由于经济和技术上的限制，仍然有必要进一步探索新的预后因素。血栓栓塞是癌症死亡的第二大原因[6]，肿瘤本身可通过多种机制激活凝血功能[7]。一方面，有报道证实在血液系统肿瘤中促凝血因子如组织因子（TF）水平增加[8]。TF可能通过刺激血管生成和转移促进肿瘤的发生和进展[9]–[10]。另一方面，炎性细胞因子、促血管生成因子和其他能够激活促凝物质释放的介质可使肿瘤细胞更易黏附于内皮细胞[7]。此外，肿瘤微环境中血小板的聚集及凝血酶、FⅦ和TF上调可使肿瘤细胞更易逃避免疫监视[11]。

DD是纤维蛋白降解的最终产物，有研究显示其为许多实体瘤的预后标志物[12]–[13]，在血液系统肿瘤中也有相关研究[14]–[15]。APTT是一项检测内源性凝血因子缺乏的敏感指标。血液内的肿瘤细胞刺激机体产生促凝物质，导致凝血酶原活化，催化纤维蛋白的形成和降解。该过程消耗了大量的凝血因子，导致DD的聚积，也引起APTT延长。

Mohren等[16]发现侵袭性淋巴瘤患者血栓栓塞并发症的发生率显著高于惰性淋巴瘤。因此侵袭性淋巴瘤患者早期诱导治疗时可进行预防性抗凝治疗。但对于血液系统疾病患者，因其本身通常伴有血小板减少，抗凝治疗通常具有较高的出血风险。因此，根据临床危险因素和生物标志物准确估计每例患者的血栓形成风险非常重要。

本研究回顾性分析了329例初诊CLL患者，结果表明高水平APTT和DD与患者TTFT和OS时间缩短相关。在此基础上，我们提出了一种新的预后模型——CSI，CSI较高的患者具有较差的TTFT和OS。而CSI联合CLL-IPI能更好地预测CLL患者的OS。

总之，APTT和DD水平高的CLL患者TTFT、OS差。而由这两项指标组成的CSI联合CLL-IPI可以提高后者对OS的预测能力。但由于本项研究为回顾性研究且纳入病例仅来源于单中心，可能存在偏倚，未来仍需多中心和前瞻性研究验证相关结论。
